# Single and combined toxicity of tadalafil (Cilais) and microplastic in Tilapia fish (*Oreochromis niloticus*)

**DOI:** 10.1038/s41598-024-64282-3

**Published:** 2024-06-25

**Authors:** Mahmoud S. Sabra, Alaa El-Din H. Sayed, Shaimaa K. A. Idriss, Hamdy A. M. Soliman

**Affiliations:** 1 Pharmacology Department, Faculty of Veterinary Medicine, Assiut University, Assiut, 71516 Egypt; 2 Zoology Department, Faculty of Science, Assiut University, Assiut, 71516 Egypt; 3 Molecular Biology Research and Studies Institute, Assiut University, Assiut, 71516 Egypt; 4 Department of Fish Disease and Management, Faculty of Veterinary of Medicine, Assiut University, Assiut, 71516 Egypt; 5 Department of Zoology, Faculty of Science, Sohag University, Sohag, 8562 Egypt

**Keywords:** Cilais, Interleukin-6, Nile tilapia, Microplastics, Blood cells, GSH, Biochemistry, Zoology, Biomarkers

## Abstract

The joint impact of tadalafil (Cilais) as a pharmaceutical residue and microplastics on fish is not well comprehended. The current study examined haematological, biochemical, and antioxidant parameters, along with immunohistochemical and histological indications in tilapia (*Oreochromis niloticus*) after being exposed to tadalafil, polyethylene microplastics (PE-MPs), and their mixtures for 15 days. The fish were distributed into 1st group control group (The fish was maintained in untreated water without any supplements); 2nd group exposed to 10 mg/L PE-MPs;3rd group exposed to 20 mg/l tadalafil (Cilais); 4th group exposed to 20 mg/l tadalafil (Cilais) + 10 mg/LPE-MPs (in triplicate). The levels of creatinine, uric acid, glucose, AST, ALT, and albumin in fish treated with tadalafil alone or in combination with PE-MPs were significantly higher than those in the control group. Fish exposed to PE-MPs, tadalafil, and tadalafil plus PE-MPs showed significantly lower levels of RBCs, Hb, Ht, neutrophils, and lymphocytes compared to the control group. Serum levels of total antioxidant capacity and reduced glutathione (GSH) were notably lowered in fish groups subjected to PE-MPs, tadalafil, and tadalafil + PE-MPs combinations in comparison to the control group. Malondialdehyde (MDA) serum levels were notably elevated in fish groups subjected to PE-MPs, tadalafil, and tadalafil + PE-MPs combinations compared to the control group. The most severe impact was observed in the tadalafil + PE-MPs combination group. Interleukin-6 (IL-6) levels were significantly increased in liver tissues following exposure to both tadalafil and microplastics compared to tissues exposed to only one substance or the control group. Changes in the gills, liver, and renal tissues were seen following exposure to PE-MPs, tadalafil, and tadalafil + PE-MPs combination in comparison to the control group of fish. Ultimately, the mixture of tadalafil and PE-MPs resulted in the most detrimental outcomes. Tadalafil and PE-MPs exhibited showed greater adverse effects, likely due to tadalafil being absorbed onto PE-MPs.

## Introduction

Due to their widespread occurrence in the ecosystem, microplastics (MPs) are a problem that affects the entire world^1^. Plastic manufacturing has increased one 100-fold to around 280 million tonnes per year worldwide, the majority of which is destined for throwaway usage^2^. Plastics are widely used in many different applications, such as plastic furniture, national sports and entertainment, construction and building materials, electrical procedures, and food packaging^3^. Most plastics' physical properties, such as chemical solidity and slow metabolic rates, along with large manufacturing rates, result in a buildup of plastic residues in the environment, causing harmful impacts in aquatics^4^.

The main sources of MPs in freshwater have been identified as surfactant use, biodegradation of large microplastics in water, and the release of wastewater from treatment facilities for sewage^5,6^. Furthermore, sewage treatment plant wastes are the leading source of freshwater environmental microplastics^7^. Freshwater may include many MPs; however, few research has been conducted to compare MPs in freshwater to those in marine water^8^. Large amounts of microplastics are constantly entering waterways through wastewater discharge^9^. In aquatic environments, microplastics can lead to behavioral abnormalities, immunological and neurological damage, hormonal interruption, biochemical disturbance, and oxidative cellular damage^10–17^. Recent research indicates that the liver is the organ most impacted in fish exposed to microplastics^19^.

The microplastic polymers that were most commonly found were polyethylene (73.08%), polypropylene (21.15%), and polystyrene (5.77%). An affirmative link was observed between the quantities of microplastics and the weight and length of fish. Microplastics were linked to human health concerns that ranged from low to moderate toxicity^18^. In freshwater fish, microplastics exposure may lead to an excessive formation of reactive oxygen species (ROS) in the mitochondria of fish hepatocytes. ROS induces the upregulation of toll-like receptor 2 and triggers the activation of downstream molecules myeloid differentiation factor 88 and tumor necrosis factor receptor-associated factor 6 in the toll-like receptor 2 signaling pathway, resulting in the phosphorylation of nuclear factor kappa B^19^.

Pharmaceuticals, or medications used in the treatment and prevention of established diseases, are the most common type of environmental contamination, causing serious health consequences. These contaminants may enter the environment at rates greater than the ecosystems' inherent capability for detoxification^20^. Furthermore, sewage treatment facilities were unable to eradicate these compounds because they may convert to metabolic by-products that were frequently more dangerous than the original compounds; as a result, they entered rivers and could spread into drinking water sources^21^.

Tadalafil, being a hydrophobic organic molecule, exhibits low solubility in water, making it challenging to accurately detect its concentration in the aqueous phase. Moreover, inadequate or absent connections between environmental concentrations and the levels of accumulation in aquatic species can result in an underestimating of the potential danger^22^. Tadalafil and other phosphodiesterase-5 inhibitors were initially created to treat angina pectoris. Later, it is utilized to treat asthma and sexual dysfunction^23^. Tadalafil, an emerging polluter, might be eliminated in stool or urine and carried through sewers to public wastewater treatment facilities alongside other pharmaceutical drugs^21^. Despite the fact that the freshwater rotifer (*Brachionus calyciflorus*) was not acutely adversely affected by tadalafil up to the highest dosage (20 mg L^−1^).Few studies have explored sildenafil residues in the environment using multi-residue approaches^24–26^, and even fewer have measured tadalafil and vardenafil residues or included their metabolic products^27,28^.

A few research have described the interaction between microplastics and medicines; according to Zhang, Ding^29^, after 14 days of exposure, roxithromycin accumulates more easily in different tissues of red tilapia exposed to MPs. In other study, transcriptome study of red tilapia exposed to MPs and either sulfamethoxazole or the beta-blocker propranolol demonstrated that MP ageing mostly influenced the expression of genes involved in the processes of metabolism, immunity, and genetic information^30^.

The Nile tilapia (*Oreochromis niloticus*) may be found in a variety of tropical and subtropical habitats across the world^31^. Furthermore, because of its tolerance to stress, it is commonly used as a model in ecotoxicology research^32,33^. According to the prior information, to the best of our knowledge, no research on the toxicity of microplastic and tadalafil combination in freshwater tilapia (*Oreochromis niloticus*) is available. Therefore, the goal of this investigation was to ascertain the harmful effects of microplastic and/or tadalafil in freshwater tilapia fish (*Oreochromis niloticus*) using several biomarkers.

## Material and methods

### Chemicals

Polyethylene microplastics (PE-MPs) powder was supplied by Toxemerge Pty Ltd. (Melbourne, Australia). Purified water (Milli-Q) was used to create the base solution from the powder in accordance with the manufacturer's instructions and kept at 4 °C in the dark until characterization^15^. AK Scientific, Inc., Union City, CA, USA, provided tadalafil (Cilais) substance.

### Justification for drug dosage selection

The dosage of tadalafil employed in our study was chosen based on previous research^21^. However, a study by Bianco et al. [^21^] indicated that there is no human health risk for exposure to sildenafil (LC50 = 42.74 for *B. calyciflorus*) and tadalafil (LC50 = no effect up to 20 for *B. calyciflorus*). However, they require special care as this form of contamination is found in any aquatic system evaluated. Another study found that the process of chlorination of sildenafil (LC50 = 0.13–0.27) and tadalafil (LC50 = 0.15–0.29) can be hazardous to fish that are exposed to it^34^. Therefore, we chose to utilize the maximum dosage of 20 mg/l of tadalafil in our research. Conversely, the PEMPs used in our investigation were selected based on earlier research^15^.

### Experimental design

Fish (*Oreochromis niloticus*) (mean body weighing 242.7 ± 22.4 g; mean body length 27.3 ± 1.4 cm) were originated from private fish farm, Assiut, Egypt. They were healthy and parasite-free according to^35^. *Oreochromis niloticus* were acclimatized for 4 weeks in 96 L glass aquaria (80 × 40 × 30 cm) filled with dechlorinated water in the laboratory conditions: conductivity (261 ± 5.2 mM cm^−1^),water temperature (26 ± 1 °C), dissolved oxygen (7 ± 0.2 mg L^−1^), pH (7.4 ± 0.4), and photoperiod 12:12 h. *Oreochromis niloticus* were fed with a commercial fish pellet feed (containing 30% protein and 12% lipid; El Nasr Company, Egypt) at rate of 3% body biomass daily. One hundred and twenty fish (*O. niloticus)* were selected and divided into four treatments, each treatment contains 30 fish in triplicates (as 10 fish for each) in 96L glass aquarium water. The fish were distributed into 1st group control group (fish exposed to only water); 2nd group exposed to 10 mg/LPE-MPs^15^; 3rd group exposed to 20 mg/l tadalafil (Cilais)^21^; 4th group exposed to 20 mg/l tadalafil (Cilais) + 10 mg/LPE-MPs. Twelve fish from each group were chosen at random after 15 days of exposure and put unconscious with crushed ice^36^. Blood samples (1.5 ml) were collected from the caudal vein, part for hematological indices, and the other part was centrifuged undercooling for biochemical and antioxidants parameters. Liver, gills, and kidney tissues were used for histological and immunohistochemical studies.

### Hemato-biochemical parameters

Hematological parameters such as the number of red blood cells (RBCs), white blood cells (WBCs), differential WBCs, blood platelets, hematocrit level (Hct) and hemoglobin level (Hb), as well as erythrocyte indices such as mean corpuscular hemoglobin (MCH), mean corpuscular volume (MCV), and mean corpuscular hemoglobin concentration (MCHC) were all measured using an automated technical analyzer (BC-2800 from Mindray).

Blood samples for biochemical parameters were obtained from fish without the use of an anticoagulant agent to acquire serum. Colorimetric determinations of the following important biochemical indices: alanine aminotransferase (ALT), aspartate aminotransferase (AST), creatinine, uric acid, albumin, globulin, and glucose were carried out according to Hamed, Soliman^15^ by using a spectrophotometer in a wavelength range of 340–546 nm (Biodiagonstic Company, Egypt).

### Antioxidant parameters and Lipid peroxidation

Reduced glutathione (GSH) concentration was evaluated according to Beutler et al.^37^. TAC (total antioxidant capacity) was determined utilizing kits (Sigma-Aldrich, USA). The malondialdehyde (MDA) level was determined using a thiobarbituric acid reaction^38^.

### Histopathological analysis

Tissue samples of the gills, liver, and kidney were collected from three fish from each group and fixed in neutral buffered formalin before being processed using a standard automated process (dehydrated through graded ethanol concentration, cleared with methyl benzoate), embedded in paraffin wax, and sectioned (5 microns)^39^. De-waxed slides are rehydrated before being stained with hematoxylin and eosin (H & E)^40^. Finally, an Olympus CH30 microscope was used to analyze and photograph the specimen. Pathology severity was graded as follows: (−), absent; (+), minor; (++), moderate; (+++), severe; and a maximum score (++++)^41^.

### Immunohistochemical analysis

An immunohistochemistry recoloring method was utilized to look at Interleukin-6 (IL-6). Four- µm -thick tissue segments were deparaffinized in xylene and rehydrated. To halt endogenous peroxidase action, the areas were submerged in 3% H_2_O_2_ for 10 min. After that, they were PBS-washed for 2 min. three times. The segments were at that point treated with standard goat serum per the prescribed procedure for 30 min at 37 °C (Vector Research facilities, Burlingame, CA), and after that for 1 h at room temperature with essential polyclonal IL-6 antibody; 1/50 (E-AB-40021, Elabscience Biotechnology Inc, USA). The expansion of polyperoxidase-anti-Mouse/Rabbit IgG is taken after 20 min. With the help of a streptavidin–biotin-peroxidase pack and Mayer's hematoxylin as a counterstain, the antigen–antibody complex was found. For each explore, positive and negative control areas were utilized. Intense brown nuclear and cytoplasmic staining assisted in identifying active IL-6 immunostaining cells. A light microscope was used to examine the slices in the 20 randomly selected locations (Olympus BX41, New York) at a magnification (50 ×). A blind fashion evaluation was carried out, with comparisons performed using the average score across all groups. The photomicrographs were taken with digital camera (canon digital camera (PowershotA95)).

### Statistical analysis

The SPSS 16.0 program for Windows (SPSS 2007, Inc., IL, USA) was used to analyze the data, and 0.05 was regarded as the point of significance. The Shapiro–Wilk test was used to determine whether the data was normal. ANOVA was used to determine the homogeneity of variances after the one-way analysis of variance (Levene’s test). Fisher’s LSD post hoc analysis was used to compare the treatment groups to the control group in the case of variance equality. Dunnett’s post hoc test was used to compare the treatment groups to the control group where there was variance inequality.

### Ethical statement

The ethics committee of the Faculty of Veterinary Medicine, Assiut University, Assiut, Egypt, authorized the experimental setup and fish handling in accordance with the OIE criteria for the use of animals in research. All methods were performed in accordance with the relevant guidelines and regulations and in accordance with ARRIVE guidelines (No, 06\2023\0037).

## Results

### Evaluation of biochemical parameters

Effects of tadalafil, PE-MPs, and their combination on the serum biochemical properties of *O. niloticus* are observed in Table 1. Fish exposed to PE-MPs, tadalafil, and tadalafil + PE-MPs combination exhibited significantly increase (*p* < 0.05) in serum creatinine, glucose levels compared to the control group. While serum uric acid and albumin levels were significantly increased (*p* < 0.05) upon fish exposure to microplastics and tadalafil + PE-MPs combination in compared to control group and these levels appeared to be in-significant (*p* > 0.05) upon exposure to tadalafil. PE-MPs and tadalafil + PE-MPs combination was the most potent treatment that affects the fish biochemical properties compared to tadalafil group. Serum globulin levels were non-significantly increased (*p* > 0.05) in tadalafil, PE-MPs, and tadalafil + PE-MPs fish groups compared to the control group. Finally, AST and ALT serum levels which is indicative of liver organ damage were significantly elevated (*p* < 0.05) in all treated-fish groups with the highest levels in tadalafil + PE-MPs combination.

**Table 1:** Effect of Microplastics and Tadalafil (Cilais) on biochemical parameters in Nile tilapia (*Oreochromis niloticus)* after exposure for 15 days.

Parameters	Control	Microplastics	Tadalafil	Microplastics + Tadalafil
Creatinine (mg/dl)	0.65 ± 0.01^a^	0.77 ± 0.01^b^	0.71 ± 0.01^c^	0.85 ± 0.02^d^
Uric acid (mmol/l)	12.6 ± 0.1^a^	13.6 ± 0.1^b^	12.7 ± 0.1^a^	15.1 ± 0.2 ^c^
Glucose (mg/dl)	98.3 ± 0.3^a^	110.3 ± 1.4^b^	103.9 ± 1.6 ^c^	117.1 ± 1.8^d^
Albumin (g/dl)	1.1 ± 0.03^a^	1.5 ± 0.03^b^	1.2 ± 0.02^a^	1.7 ± 0.04^c^
Globulin (g/dl)	2.2 ± 0.01^a^	2.2 ± 0.02^a^	2.3 ± 0.03^a^	2.3 ± 0.04^a^
AST (µ/l)	56.0 ± 0.3^a^	65.9 ± 0.9^b^	60.5 ± 0.4^c^	70.4 ± 0.7^d^
ALT (µ/l)	29.3 ± 0.7^a^	36.4 ± 0.4^b^	33.3 ± 0.6^c^	40.0 ± 0.7^d^

Data are represented as means ± SE. Values with different superscript letter in the same row for each parameter are significantly different (*P* < 0.05).

### Evaluation of hematological parameters

Table 2 showed that RBCs, Hb, Ht (PCV) and neutrophils were significantly decreased (*p* < 0.05) in fish groups exposed to PE-MPs, tadalafil, and tadalafil + PE-MPs combination compared to the control group. Tadalafil + PE-MPs combination group showed the highest toxic effects on fish blood profile followed by PE-MPs and tadalafil compared to the control group (Table 2). In other hand, MCV, lymphocytes levels were significantly increased (*p* < 0.05) in all fish treated groups with highest levels. With tadalafil + PE-MPs combination group compared to the control group (Table 2). While MCH, MCHC, monocytes, and eosinophils were not statistically significant decline (*p* > 0.05) in tadalafil, PE-MPs and tadalafil + PE-MPs combination groups compared to the control group (Table 2). A significant increase (*p* < 0.05) in platelet count appeared with all treated groups except of tadalafil alone exposed fish while a significant decrease (*p* < 0.05) in neutrophils percentage in all treated groups except for microplastic treated fish (Table 2).

**Table 2:** Effect of microplastics and Tadalafil (Cilais) on hematological parameters in Nile tilapia (*Oreochromis niloticus)* after exposure for 15 days.

Parameters	Control	Microplastics	Tadalafil	Microplastics + Tadalafil
RBCs (Million/mm^3^)	1.96 ± 0.01^a^	1.76 ± 0.02^b^	1.83 ± 0.02^c^	1.69 ± 0.01^d^
Hemoglobin (Hb) (g/dl)	9.28 ± 0.08^a^	8.03 ± 0.01^b^	8.73 ± 0.06^c^	7.68 ± 0.08^d^
Ht (PCV) (%)	33.11 ± 2.4^a^	24.25 ± 0.5^b^	25.63 ± 0.2^b^	23.38 ± 0.2^b^
MCV (µm^3^)	120.8 ± 2.3^a^	132.6 ± 2.9^b^	131.5 ± 1.9^b^	137.0 ± 1.5^b^
MCH(Pg)	47.32 ± 0.2^a^	48.6 ± 0.6^a^	48.6 ± 0.6^a^	46.7 ± 0.5^a^
MCHC (%)	36.8 ± 0.5^a^	37.3 ± 1.1^a^	36.9 ± 0.4^a^	35.9 ± 0.4^a^
Platelets (Thousands/mm^3^)	314.8 ± 1.7^a^	325.8 ± 1.7^b^	313.5 ± 0.9^a^	333.8 ± 4.6^b^
WBCs (Thousands/mm^3^)	844.3 ± 3.5^a^	841.0 ± 3.7^a^	847.5 ± 4.9 ^a^	845.0 ± 3.2^a^
Neutrophils (%)	6.8 ± 0.3 ^a^	5.8 ± 0.3^ab^	5 ± 0.4^b^	5 ± 0.4^b^
Lymphocytes (%)	88 ± 0.4^a^	90.3 ± 0.5^b^	91.3 ± 0.3^b^	92.00 ± 0.6^b^
Monocytes (%)	3.3 ± 0.3^a^	3 ± 0.4^a^	2.8 ± 0.3^a^	2.5 ± 0.3^a^
Eosinophils (%)	2 ± 0.0^a^	2 ± 0.0^a^	2 ± 0.0^a^	1.5 ± 0.3^a^

Data are represented as means ± SE. Values with different superscript letter in the same row for each parameter are significantly different (*P* < 0.05).

### Evaluation antioxidant parameters and Lipid peroxidation

Table 3 demonstrated that total antioxidant capacity (TAC) and reduced glutathione (GSH) serum levels were significantly decreased in fish groups exposed to PE-MPs, tadalafil and tadalafil + PE-MPs combination compared to the control group. Tadalafil + PE-MPs combination showed the highest toxic effects on fish antioxidant parameters followed by PE-MPs and tadalafil compared to the control group (Table 3). In other hand, malondialdehyde (MDA) serum levels were significantly increased (*p* < 0.05) in fish groups exposed to PE-MPs, tadalafil and tadalafil + PE-MPs combination compared to the control group with the highest harmful effect with tadalafil + PE-MPs combination group (Table 3).

**Table 3:** Effect of microplastics and Tadalafil (Cilais) on antioxidant and oxidant parameters in Nile tilapia (*Oreochromis niloticus)* after exposure for 15 days.

Parameters	Control	Microplastics	Tadalafil	Microplastics + Tadalafil
Total antioxidant capacity (TAC) (μM/L)	1.03 ± 0.01^a^	0.91 ± 0.00^b^	0.95 ± 0.02^b^	0.88 ± 0.03^b^
Reduced glutathione (GSH) (µmol/dL)	4.3 ± 0.1^a^	3.5 ± 0.02^b^	3.6 ± 0.1^b^	3.4 ± 0.1^b^
Malondialdehyde (MDA) (nmol/ml)	10.8 ± 0.1^a^	11.5 ± 0.1^b^	11.2 ± 0.03^cb^	12.04 ± 0.1^d^

Data are represented as means ± SE. Values with different superscript letter in the same row for each parameter are significantly different (*P* < 0.05).

### Interleukin-6 (IL-6) immunohistochemistry alterations in fish liver

**Figure 1:**
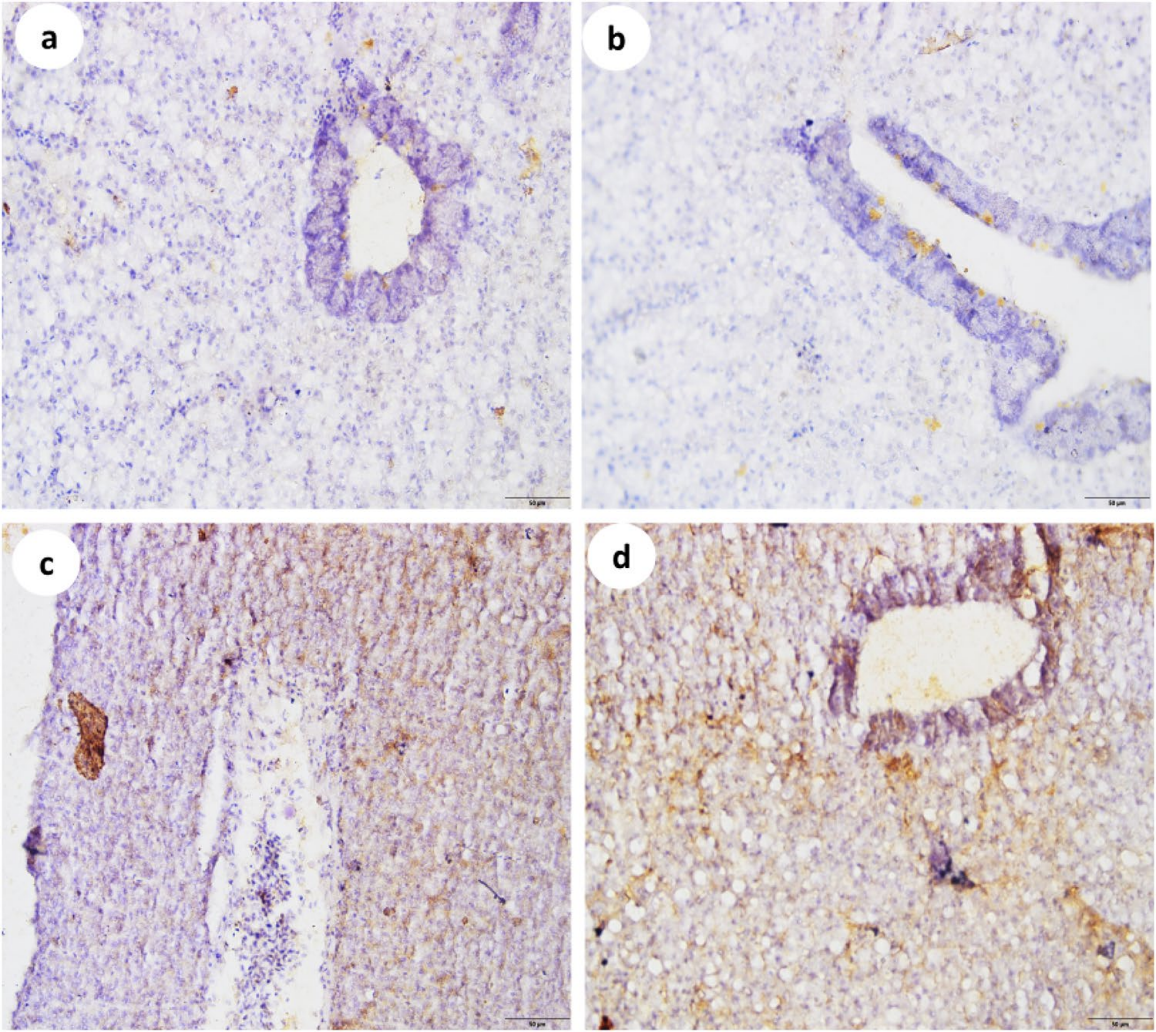
Interleukin-6 immunoreactivity in the liver tissues of Nile tilapia (*Oreochromis niloticus*). (**a**) Control group, (**b**) microplastic exposed group, (**c**) tadalafil exposed group, (**d**) tadalafil + microplastics co-exposure.

Results of the IL-6 immunoreactivity are shown in Fig. 1. Relatively little active IL-6 immunoexpression was seen in the liver tissue of the control group (Fig. 1a). Fish treated with microplastics exhibited little IL-6 immunoexpression (Fig. 1b). IL-6 immunoreactivity was moderate in tilapia fish subjected to tadalafil (Fig. 1c). IL-6 immunoreactivity was highly elevated in liver tissue after co-exposure to tadalafil and microplastics combination (Fig. 1d) compared to liver tissue after single exposure.

### Histopathological analysis

**Figure 2:**
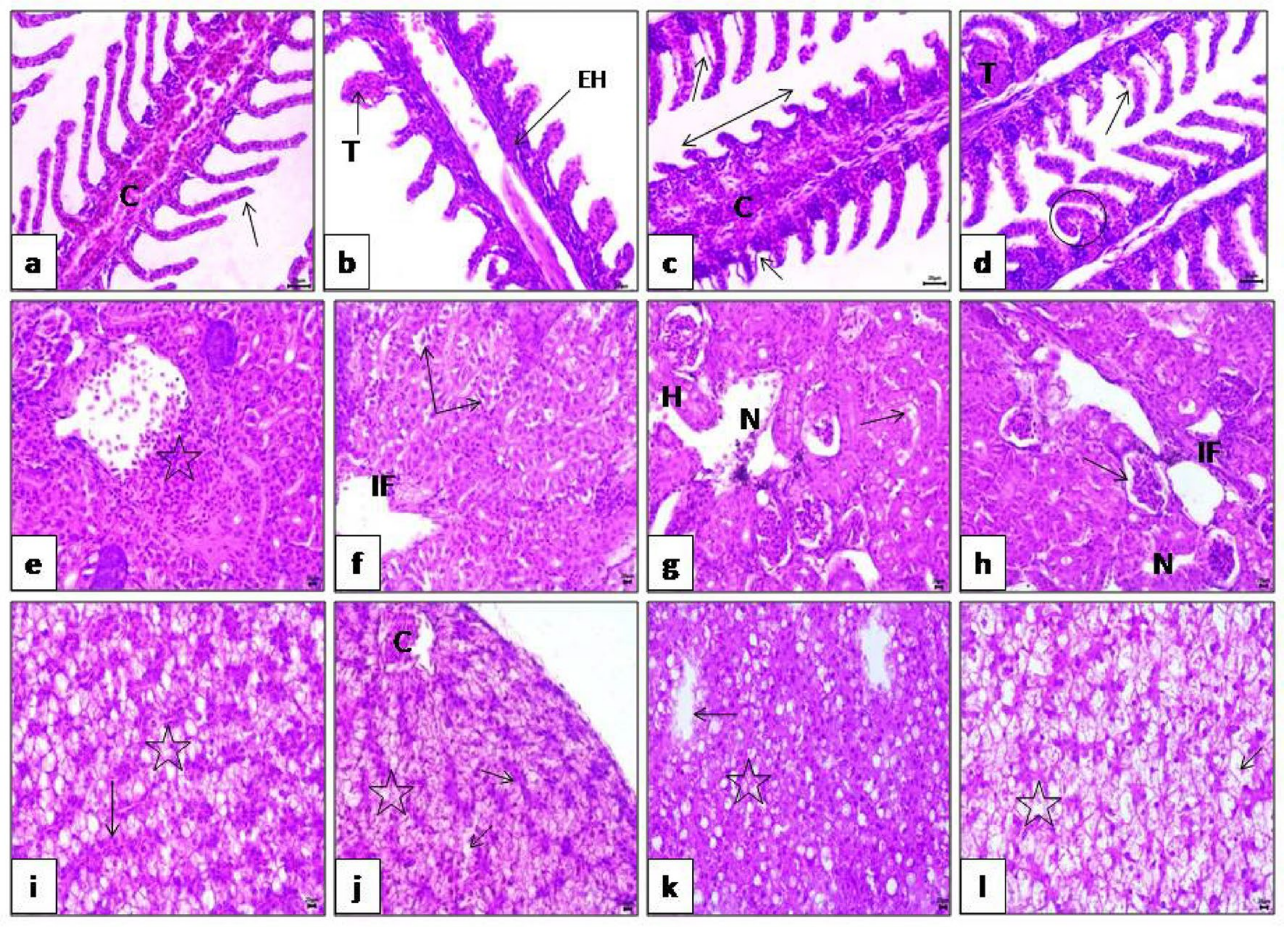
(**a**) Gills after exposure to tadalafil group showing congestion of primary lamellae (C) and mild curling of secondary lamellae (arrow). (**b**) Gills after exposure to microplastic group showing telangictasis (T) of secondary lamellae with epithelial hyperplasia (EH) of primary gill lamellae. (**c**, **d**) Gills after exposure to tadalafil microplastic group showing telangictasis (T), sever curling (circle), shortening of secondary lamellae (double arrow) and epithelial lifting (arrow) of secondary lamellae. (**e**) Kidney after exposure to tadalafil group showing thrombus (star). (**f**) Kidney after exposure to microplastic group showing perivascular inflammatory cell infiltration (IF) and vacuolar degeneration (arrow) of renal tubules. (**g**, **h**) Kidney after exposure to tadalafil and microplastic group showing sever necrosis of hemopiotic tissue (N), perivascular inflammatory cell infiltration (IF), dilatation of Bowman’s space (arrow) hemorrhages (H) and vacuolar degeneration (arrow) of renal tubules. (**I**) Liver after exposure to tadalafil group showing vacuolar degeneration (star) and congestion of sinusoids (arrow). (**j**) Liver after exposure to microplastic group showing congestion of blood vessels (C) and sinusoids (arrow), vacuolar degeneration (star) and mild hepatic necrosis (dashed arrow). (**k**, **l**) Liver after exposure to tadalafil microplastic group showing sever necrosis (arrow) and sever vacuolar degeneration of hepatocytes (star). (**h**, **E** × 400).

Tadalafil exposure caused modest secondary lamellae curling and primary lamellae congestion in gills (Fig. 2a). Gills that had been exposed to microplastic showed secondary gill lamellae telangiectasia together with primary gill lamellae epithelial hyperplasia (Fig. 2b). Additionally, after exposure to tadalafil and microplastics in combination, telangiectasia, curling, and shortening of secondary lamellae with epithelial lifting were clearly seen to a severe degree (Fig. 2c, d).

After being exposed to tadalafil, kidney tissues exhibited thrombus (Fig. 2e). Kidney after exposure to microplastic revealed vacuolar degeneration of renal tubules and perivascular inflammatory cell infiltration (Fig. 2f). Furthermore, the kidney revealed severe necrosis of hematopoietic tissue, perivascular inflammatory cell infiltration, dilatation of the Bowman’s space, hemorrhages, and vacuolar degeneration of the renal tubules after exposure to the tadalafil and microplastic combination (Fig. 2g, h).

Vacuolar degeneration and sinusoidal congestion were seen in our liver observations following exposure to tadalafil (Fig. 2 i). After exposure to microplastics, the liver exhibited vacuolar degeneration, blood vessel and sinusoidal congestion, and mild hepatic necrosis (Fig. 2j). Additionally, the liver showed significant necrosis, congestion of the hepatopancreas, and vacuolar degeneration of the hepatocytes after exposure to tadalafil and microplastic (Fig. 2k, l). In Table 4, the frequencies of the modifications were listed.

**Table 4:** Effect of microplastics and Tadalafil (Cilais) on histopathological scoring of gills, kidney and liver in Nile tilapia (*Oreochromis niloticus)* after exposure for 15 days.

Lesions	Tadalafil	Microplastic	Tadalafil and Microplastic
Gills			
Lamellar congestion of primary lamellae	++	+	++++
Epithelial hyperplasia of primary lamellae	−	+++	++++
Epithelial necrosis and degeneration of primary lamellae	++	+++	++++
Separation of primary lamellae from basement membrane	++	−	+++
Lamellar congestion of secondary lamellae	−	++	+++
Secondary lamellar telangictasis	−	+	+++
Epithelial detachment of secondary lamellae	−	+	++
Curling of secondary lamellae	++	+++	++++
Shortening of secondary lamellae	+	++	+++
Fusion of adjacent secondary lamellae	−	++	+++
Kidney			
Dilatation of bowman’s space	+	++	++++
Coagulative necrosis of tubular epithelium	+	++	+++
Vacuolar degeneration of tubular epithelium	++	+++	++++
Necrosis of hemopiotic tissue	+	++	+++
Inflammatory cell infiltration	+	+++	++++
Degeneration of blood vessels wall	+	+	+++
Interstitial hemorrhage	++	+	++
Thrombus	+	++	+++
Liver			
Hepatic cord disarray	++	+++	++++
Vacuolar degeneration of hepatocytes	+++	++	++++
Necrosis of hepatocytes	++	+++	++++
Congestion of blood vessels	+	+++	+
Degeneration and necrosis of sinusoids	++	++	+++
Congestion of sinusoids	++	+++	+
Congestion of hepatopancreas	++	+	+++

Severity of pathology was evaluated as: (−), absent; (+), minimal; (++), moderate; (+ + +), severe; a maximum score (++++).

## Discussion

Plastics, both large and small-scale, are endangering people's lives and the natural ecosystem's health. The volume of plastic waste in macro, micro, and nano sizes is anticipated to double by 2030^42^. More importantly, because wastewater treatment facilities are one of the primary sources of microplastics in freshwater rivers, where discharged medicines come into contact with microplastics and form microplastic-pharmaceutical complexes, these complexes are toxic to aquatic creatures if they consume them^43^. Additionally, MPs can operate as carriers of organic pollutants, including pharmaceuticals, boosting their bioaccumulation and biomagnification up the food chain when consumed by animals. This is because MPs have the capacity to adsorb a range of organic pollutants^44,45^. The current study’s findings revealed increased serum levels of albumin, glucose, AST, ALT, uric acid, and creatinine in Nile tilapia fish groups exposed to both microplastics and tadalafil + PE-MPs combination, with higher significant levels in the combination group, whereas serum albumin and uric acid levels did not change after fish exposure to tadalafil. Several studies on the toxicity of microplastics on several fish species found an increase in albumin, creatinine, glucose, and uric acid concentrations^15,46–50^.

Fish kidneys operate as a hemopoietic organ, a waste elimination system, and a regulator of the osmotic pressure between the fish and its surroundings^51^. Microplastics have been shown to accumulate in the liver, kidney, and gut of fish, causing inflammation and organ damage^52^. In addition, uric acid and creatinine can be utilized to detect renal filtration rate and as a diagnosis for renal insufficiency^53^. As a result, the present study’s findings point to kidney impairment, particularly in the tadalafil and PE-MPs combination group.

Glucose may be an imperative metabolic fuel required for the proper operation of vitality related capacities within different organs. Glucose is assembled in reaction to changes in physiological or natural circumstances^54,55^. Elevated serum glucose levels imply glycogen breakdown in hepatic tissue or a decline in glucose absorption^56^. Similarly, to the current work, Nematdoost Haghi and Banaee^49^ discovered that single or combination of paraquat and MPs increased sugar levels in common carp (*Cyprinus carpio*) to overcome the energy shortage. Another study discovered that hyperglycemia caused by microplastics was associated with increased gluconeogenic activity and cortisol production following fish stress exposure^57^.

The protein albumin is in charge of moving fatty acids and steroid hormones from fat cells to muscle. It is in charge of around 80% of all osmotic control^58^. Moreover, albumin levels are expected to rise due to breakdown in hepatic and renal tissues^15,59^. Espinosa, Cuesta^47^ also discovered that an increase in albumin plasma concentrations suggests that MPs may harm the liver or kidney tissues. As a result, the present study’s findings point to greater blood albumin and glucose levels, particularly in the tadalafil and PE-MPs combination group, which may be linked to eventual tissue damage and energy crisis compensation in this study. In addition, after concurrent exposure to MPs and bacteria (*Yersinia ruckeri*), the lipid profile, total protein, albumin, and globulin reduced, although glucose, liver, and kidney functions increased in the plasma of fish rainbow trout (*Oncorhynchus mykiss*)^60^.

Alanine transaminase is an enzyme present in the liver that helps transform proteins into energy for the liver cells. When the liver is injured, ALT is released into the circulation, where it accumulates. Furthermore, aspartate transaminase is an enzyme that aids in the metabolism of amino acids. AST, like ALT, is generally present in low amounts in the blood. A rise in AST values might suggest liver damage, illness, or muscle injury^61,62^. According to Kumar, Sharma^63^, AST and ALT enzymes are found in cells of several organs of the body systems. The release of these enzymes and their increased levels in the blood are thought to be indicators of cell membrane injury^15^. According to one study, hepatotoxicity caused by microplastics results in the liberation of intracellular enzymes^64^, while another study found that an increased enzyme activity (AST and ALT) in the fish gill, liver, and kidney are designed to boost the function of proteins in energy synthesis during toxicant stress^65^. Also, following exposure to MPs and/or pyrene, common goby (*Pomatoschistus microps*) showed changes in biochemical markers^50^ as well as to MPs and/or nickel^66^. Additionally, the activity of ALT, AST and ALP increased in response to paraquat and/or MPs, which may imply injury to common carp *(Cyprinus carpio*) organ plasma membranes^49^. Moreover, after administering tadalafil, male albino wistar rats showed significantly higher serum levels of AST, ALT, ALP, total bilirubin, and unconjugated bilirubin^67^.

Microplastic toxicity in fish cells, according to research, is mostly caused by oxidative stress, which involves redox imbalance disturbance, cellular component destruction, and high production of ROS. Furthermore, free radicals produced by MPs exposure are eliminated by an antioxidant system that includes GSH and the enzymes that depend on it. As a result, GSH could be utilized as a marker to examine fish's responses to antioxidants after being exposed to MPs^68^. In addition, oxidative stress may develop when fish are exposed to harmful substances like MPs because of increased ROS production. Malondialdehyde is utilized as a diagnostic for lipid peroxidation since it is the end product of oxidative damage to lipids^69^. Oxidative stress, which can cause inflammatory processes and suicide of cells, is created when ROS levels in fish tissues are elevated^10^. Based on previous findings, the results of the present study showed a considerable rise in malondialdehyde levels and a fall in TAC and glutathione levels, particularly with the tadalafil-PE-MPs combination, which was reflected by organ damage in histopathological and immunohistochemistry findings. In accordance with our results, according to Fonte, Ferreira^70^, juveniles of the common goby (*Pomatoschistus microps*) subjected to the combination treatment including MPs and the highest cefalexin concentration showed increases in acetylcholinesterase activity and lipoperoxidation. It was shown that the content of superoxide dismutase (SOD) and MDA in the liver of a loach (*Misgurnus anguillicaudatus*) were elevated by venlafaxine and O-desmethylvenlafaxine^71^. However, MDA levels dropped, and SOD activity considerably increased in the co-exposure treatments of MPs and roxithromycin compared to roxithromycin alone, demonstrating that oxidative damage in the presence of MPs and roxithromycin was reduced in the liver of red tilapia^72^. In fish intestines, MPs alone increased SOD and LDH activity; however, co-exposure to tetracycline-MPs decreased oxidative stress^73^. The hepatocytes of rainbow trout exposed to MPs and bacteria (*Yersinia ruckeri)* concurrently showed increased SOD activity and malondialdehyde levels, while catalase activity, glutathione peroxidase, and total antioxidant levels were all reduced when dietary MPs were paired with a bacterial challenge^60^. Sahabuddin, Noreen^74^ mentioned that the combination of (MPs + oil + Corexit) disrupts catalase, and glutathione S-transferase levels in Asian seabass (*Lates calcalifer*).

The current study's hematological effects demonstrated a substantial drop in erythrocyte count, blood Hb, and PCV following exposure to tadalafil, PE-MPs, and their combinations, with the tadalafil + PE-MPs exposed fish group showing the greatest reduction. On the other hand, MCV, lymphocytes, and neutrophils percentages were considerably greater in the combination treatment group. Toxic substances have a negative influence on the oxygen-carrying capability and electrolyte balance of the blood, resulting in a decrease in cell size due to RBC exosmosis^75^. Microplastics caused anemia in a number of aquatic species and decreased hematological traits in fish. This is thought to be because stress and/or toxic substances slowed down the rate of hemoglobin biosynthesis, which led to tissue oxygenation disorder^68,76^. Furthermore, the percentages of RBCs, basophils, thrombocytes, and eosinophils reduced following exposure to the combination of (MPs + oil + Corexit) in Asian seabass (*Lates calcalifer*)^74^.

Microplastics are recognized as foreign entities capable of stimulating or inhibiting immune function via immunotoxicity, meaning that MPs can influence fish immunity in a number of ways. Similarly, multiple investigations have demonstrated that MP (Polystyrene and PE) exposure boosts fish immune responses, such as lysozyme and neutrophil numbers^77,78^. The chemokine interleukins have been shown to predominantly attract the migration of neutrophils, T-lymphocytes, and basophils in the body^79,80^ and this could also be attributed to an increase in both neutrophils and lymphocytes levels in the current study as a result of significantly higher levels of liver IL-6 expression, particularly in fish exposed to tadalafil-PE-MPs combination. IL-6 is a pro—inflammatory cytokine that has been found to be a key factor in hemostasis, to have pro- and anti-inflammatory effects in fish, and to be important in the humoral immune response in Nile tilapia fish^81,82^. Furthermore, during the initiation of the acute phase response, IL-6 is assumed to be important for signaling hepatocytes to produce acute phase proteins. It is usually considered that IL-6 enters the liver either endocrinely through immune cells at the site of injury or paracrinely through hepatic immune cells within the liver^83^. Our reported histological findings are supported by the immunohistochemical analysis of IL-6 in liver tissues that was prominently elevated in the microplastic- tadalafil combination group and to a smaller degree in the individual treatment fish group in the current study, implying more liver tissue damage in the combination group and the IL-6 elevation may be a value in the liver tissue repair process. In the same manner, Zhang, Tang^72^ reported that zebra fish liver immune systems produce pro-inflammatory cytokines in response to toxicity, and that their livers repair the inflammatory damage by increasing the activities of anti-inflammatory cytokines.

Histopathological alterations can be utilized as markers for the impact of various contaminants on organisms and represent the general health of the ecosystem's population^84^. Gills are the primary target organ for their respiratory, osmoregulatory, and excretory functions, as well as various physiological processes such as metabolite excretion, body fluid permeability balance, and acid–base regulation balance, all of which are sensitive to water pollution^85^. Previous studies divided gill injuries into two categories: those brought on by defense reactions, such as proliferation of the gill filament epithelium and swelling of gill lamellae, and those brought on by direct injury, such as necrosis and peeling of gill epithelium^86^.

Several changes in gill tissue were detected in this research, including congestion of main lamellae, telangiectasia, curling and shortening of secondary lamellae. In addition, following tadalafil, microplastic, and tadalafil + microplastic groups in arrangement, epithelial necrosis and degeneration of primary lamellae were found in a deteriorated way. Like our observations, Hamed, Soliman^14^ discovered dilatation and congestion of primary gill blood arteries. Also, hyperplasia of epithelial cells between secondary lamellae resulted in fusion at the basal part of the secondary lamellae, shortening and degeneration of secondary lamellae, and epithelial lifting of secondary lamellae, it agreed with other research' findings on the effects of MPs, such as the enlargement and expansion of gill lamellae and the degeneration and collapse of the apical portion of gill filaments^87–90^. Karbalaei, Hanachi^91^ discovered the most histological changes in the gills of young rainbow trout when chlorpyrifos was coupled with PS-MPs. In the gills and liver of Asian seabass (*Lates calcalifer*), exposure to MP and a combination of MP + oil resulted in tissue damages, according to Sahabuddin, Noreen^74^.

Besides excretory functions, the fish kidney maintains the osmotic equilibrium between the fish and its environment. The kidney can eliminate ultrafine particles, but MP deposition and accumulation in the kidney causes physical harm and an immunological response^92,93^. In our present study, kidney exhibited vacuolar degeneration and necrosis of tubular epithelium. Also, thrombus, diffuse necrosis of hematopoietic tissue with hemorrhages was clearly observed in all groups and to a high degree after exposure to tadalafil and microplastic combination group. In early immature discus fish, MPs exacerbated the kidney damage brought on by cadmium, as demonstrated by Wen et al.^94^. As a result, our data provide more evidence for the dangers of MPs and their adsorbed drugs. Excessive ROS generation and antioxidant system suppression are two of the most prominent causes of kidney damage^95,96^. Zhu, Chernick^97^ also found that dietary exposure to polystyrene MPs caused glomerulopathy, glomerulomegaly, and Bowman's space extension in mature Japanese medaka. The kidney showed signs of fatty tubules, enlargement of the intertubular space, entire deformation and shrinkage of the glomerulus, vacuolated glomerular cells, congested blood capillaries, inflammatory cells, as well as vacuolated, and atrophy convoluted tubules [^12^]. In addition, Espinosa et al. [^47^] noted the degeneration of renal tissue in zebrafish that had consumed MP particles. Furthermore, raising angiotensin II levels can partially raise blood pressure in people following exposure to diethylhexyl phthalate^98^.It has been determined that an increase in blood pressure places the glomerular walls under mechanical stress that may cause damage^99^.

Because one of the most essential functions of the liver is to purify blood of toxins, it is regarded as an indication of aquatic environmental contamination^100^. Our findings revealed severe liver damage, as hepatic cord disarray with clear vacuolar degeneration, hepatic necrosis, and congestion of blood vessels and sinusoids were evident in all groups and to a greater extent after exposure to tadalafil and the microplastic combination group. This result is in line with earlier results that suggest exposure to MPs may have an impact on the liver's histoarchitecture, vacuolization, passive hyperemia, and hepatic necrosis^101,102^, hydropic degeneration, dilated sinusoids^103,104^, loss of association of parenchyma, and congestion of blood sinusoids^90,105, 106^.

According to Yu, Zhang^107^, co-exposure with micro-sized plastics mitigated the intestinal damage brought on by a single oxytetracycline exposure. They demonstrated that exposure to oxytetracycline, nano-sized plastics, and their combination exposure induced intestinal epithelial damage. Mostafa-Hedeab, Hanyelhady^108^ investigated the effect of tadalafil on ischemia–reperfusion injury-induced apoptosis in the liver of rats, observing hepatic necrosis, sinusoidal congestion, Van Kupffer cell growth, and severe vacuolation of the hepatocytes. They found that tadalafil can reduce IR-induced liver damage by acting as an antioxidant, anti-inflammatory, and anti-apoptotic agent. Inhibition of protein synthesis, energy depletion, microtubule disassembly, electrochemical disturbances, modification of energy metabolism, and hepatic erythropoiesis were all linked to hepatocyte injury^46,109^.

## Conclusions

There is currently no experimental data of any type regarding the effect of tadalafil on fish or their interaction with other toxicants residue in water. This investigation revealed that exposure to MPs with tadalafil might cause oxidative stress, changes in hematological and biochemical parameters, as well as damage to various organs of fish more than each drug alone, and so have a detrimental influence on the viability and longevity of fish. Toxic compounds, drug residues, and harmful pathogens can be passed to fish through MPs and subsequently to humans. As a result, various chronic sickness outbreaks affect people. As a result, it is necessary to reduce MPs contamination, improve the shelf life of plastic objects, and raise awareness in order to severely limit the input into ecosystems and let the aquatic ecology recover.

## Data Availability

The datasets generated during and/or analyzed during the current study are available from the corresponding author on reasonable request.

## Abbreviations

PE-MPs: Polyethylene microplastics
TAC: Total antioxidant capacity
GSH: Reduced glutathione
MDA: Malondialdehyde
ROS: Reactive oxygen species
MPs: Microplastics
ALT: Alanine aminotransferase
AST: Aspartate aminotransferase
RBCs: Red blood cells
WBCs: White blood cells
Hct: Hematocrit level
Hb: Hemoglobin level
MCH: Mean corpuscular hemoglobin
MCV: Mean corpuscular volume
MCHC: Mean corpuscular hemoglobin concentration
IL-6: Interleukin-6
